# Fracture of the Lower Jaw

**Published:** 1861-01

**Authors:** H. Howard Hayward


Fracture of the Lower Jaw.—The importance of the mode of treatment so ably carried out by Mr. James Salter, of Guy's Hospital, as reported in the *Lancet*, (*ante* p. 137), for the reduction of impacted fractures of the alveolar arch of the superior maxilla, is well worthy of the careful attention, not only of surgeon-dentists, but general surgeons. The application of such a principle to fractures, is a large extension of the plan of regulating misplaced teeth by elastic metallic pressure, which has been so long in successful operation. At the latter part of Mr. Salter's excellent paper, reference is made, not only to the much greater difficulty of applying mechanical contrivances to the fractured inferior maxilla, but there is a point which is a far more serious undertaking—viz., maintenance of the apparatus *in situ* comfortably to the patient, until the uniting lymph is firm enough to resist the traction of numerous antagonistic muscles.

The subjoined abridged account of a case read by Mr. James R. Lane to the Western Medical Society, and slightly noticed in the journals of the date, of a compound commuted fracture of the lower jaw, prior to that reported by Mr. Salter, in which the wearing of a metallic socket, fitting the teeth and gums, was not sufficient to maintain the broken ends in exact juxtaposition, on account of the obliquity of the fracture and antagonism of the muscles, perhaps may prove of value to surgery at some future period:—

John S——, aged thirty-two, was admitted into St. Mary's Hospital, under the care of Mr. James Lane, September, 1858. He had sustained several injuries about the head and neck by a fall from a cart, the wheel of which passed over his head, and fractured the lower jaw at the left of the symphysis, between the central and lateral incisors, obliquely downwards and outwards. The left portion was depressed about three quarters of an inch, and overlapped by the right. A large spiculum of bone on the right side, including two teeth, was also loose; but the fracture here did not extend to the lower margin After the failure of all the contrivances, including Lonsdale's apparatus, Mr. James Lane, hoping to modify Lonsdale's splint, called my attention to it, as the setting of the fracture could not be maintained by any of the apparatuses. With the assistance of others the fracture was reduced, and retained *in situ* long enough to take a model in beeswax. A silver plate was made to cap and fit the teeth and the gums before and behind them for a short distance; but I found that this did not keep the fractured ends in accurate apposition, as the spasmodic action of the muscle of the left side of the maxilla gradually jerked up the plate, though it fitted accurately. To meet this difficulty, I soldered to the upper surface of the plate, at each side, a doubly-curved silver rod, each of which projected out of his mouth at the angles, and was tied to a light gutta-percha splint beneath the chin. This placed him in a comparative state of comfort, immediately enabling him to feed himself and articulate without pain.

At the fourth week the splint was removed and the silver rods were cut off. The following week he left the hospital, with the plate in; the "bite" was perfectly accurate, and the position was well maintained by the tempcrary callus.

I accidentally saw him a few months afterwards; he had worn the plate for two or three weeks immediately on leaving the hospital, and subsequent to that only at night. The line of fracture was not detectable; but one of the teeth, where the supperation of the periosteum had been most extensive, had necrossed and required removal.

[*Lancet.*] H. Howard Hayward, M. R. C. S.

